# A Potential Mechanism for the Anti-Apoptotic Property of Koumine Involving Mitochondrial Pathway in LPS-Mediated RAW 264.7 Macrophages

**DOI:** 10.3390/molecules21101317

**Published:** 2016-10-01

**Authors:** Zhi-Hang Yuan, Zeng-Enni Liang, Jing Wu, Jin-E Yi, Xiao-Jun Chen, Zhi-Liang Sun

**Affiliations:** 1 Department of Clinical Veterinary Medicine, College of Veterinary Medicine, Hunan Agricultural University, Changsha 410128, China; yuanzhihang84@163.com (Z.-H.Y.); wu23jing@aliyun.com (J.W.); smart16@aliyun.com (J.-EY.); s51857176@gmail.com (X.-J.C.); 2 Hunan Co-Innovation Center for Utilization of Botanical Functional Ingredients, Changsha 410128, China; 3 Department of Hunan Agricultural Product Processing Institute, Changsha 410128, China; enni_007@163.com

**Keywords:** koumine, anti-apoptotic effect, mitochondria, caspase

## Abstract

Koumine is a kind of alkaloid extracted from *Gelsemium elegans* (*G. elegans*). Benth, which has shown promise as an anti-tumor, anxiolytic, and analgesic agent. In our present study, the effect of koumine on lipopolysaccharide (LPS)-mediated RAW 264.7 cell apoptosis was evaluated. MTT assays showed that koumine obviously increased cell viability in LPS-mediated RAW 264.7 macrophages. Preincubation with koumine ameliorated LPS-medicated apoptosis by decreasing reactive oxygen species (ROS) production, which resulted in a significant decrease in the levels of nitric oxide (NO) and inducible nitric oxide synthase (iNOS). In addition, koumine-pretreated RAW 264.7 macrophages exhibited reduction of LPS-induced levels of TNF-α, IL-1β, and IL-6 mRNA. Furthermore, pretreatment with koumine suppressed LPS-mediated p53 activation, loss of mitochondrial membrane potential, caspase-3 activation, decrease of Bcl-2 expression, and elevation of Bax and caspase-3 expressions, suggesting that koumine might act directly on RAW 264.7 cells to inhibit LPS-induced apoptosis. It seems as though the mechanism that koumine possesses is the anti-apoptotic effect mediated by suppressing production of ROS, activation of p53, and mitochondrial apoptotic pathways in RAW 264 cells. Koumine could potentially serve as a protective effect against LPS-induced apoptosis.

## 1. Introduction

Apoptosis, an evolutionarily conserved form of cell death, is a pathophysiological and physiological process involved in various signaling pathways [1]. These pathways can be triggered by a host of cytotoxic stimulus, which induce activation of executioner caspases and other signaling cascades that ultimately lead to apoptotic cell death. A structural part of the outer membrane of Gram-negative bacteria called LPS involves in a variety of inflammatory disorders via regulation of the expression of a variety of inflammatory cytokines and cytotoxic mediators, and has been widely applied in animals as a model for anti-inflammatory evaluation [2]. Additionally, LPS triggers severe immunological reactions, resulting in cell apoptosis [3]. Currently, LPS-induced apoptosis has been studied both in vitro and in vivo. LPS was reported to efficiently mediate chondrocyte apoptosis evaluated by the increase of caspase-3 expression and those caspase-3 related proteins [4]. LPS stimulates apoptosis by death pathway and may utilize a novel death domain-containing protein to transduce a death signal in a human microvascular endothelial cell line, HMEC-1 [5]. During bacterial infection, acute lung injury is related with neutrophilic inflammation, disruption of the alveolar-capillary barrier, and cell apoptosis [6]. Cell apoptosis was also investigated in animal models with LPS administration. It was found endothelial cell apoptosis in the liver and lung of mice injected with LPS [7].

**Figure 1:**
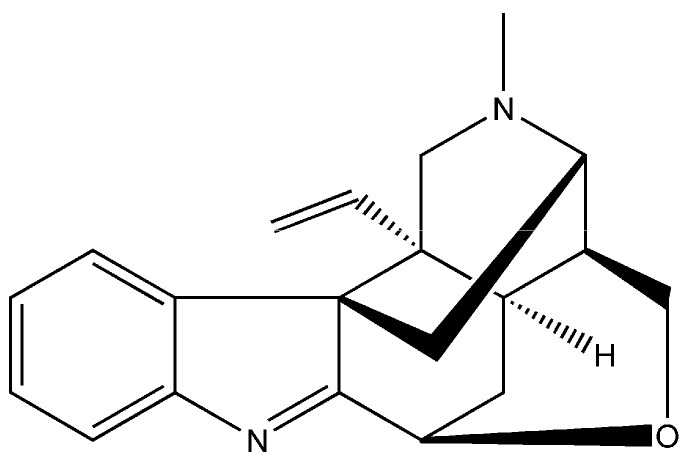
Chemical structure of koumine.

Previous investigations have shown that alkaloids extracted from Chinese medicinal plants protect against LPS-induced apoptosis. It has been proved that alkaloid-enriched extract from *Dendrobium nobile* Lindl. prevent against LPS-induced apoptosis in rat brain [8]. Lu et al. reported that total alkaloids from rhizome *Coptis chinensis* are a potent protective agent against *Helicobacter pylori* LPS-induced gastric mucosal inflammation, whose mechanisms may be related to its inhibitory effect on epithelial cell apoptosis [9]. *Gelsemium* is an Asian and North American genus of flowering plants belonging to the family Loganiaceae, which contains three commonly known species: *Gelsemium elegans* (*G. elegans*), *Gelsemium sempervirens* (*G. sempervirens*), and *Gelsemium rankinii* (*G. rankinii*). *G. elegans* grown in China and Southeast Asia was reported to produce toxicity in humans. So far, about 49 alkaloids were isolated from *G. elegans* [10]. Though these alkaloids are similar in structure, their pharmacological action are not the same. Interestingly, some alkaloids extracted from *G. elegans* can increase the efficiency of animal growth. For example, extended oral administration with low-dose koumine can help growth promotion in pigs. Koumine (molecular formula: C_20_H_22_N_2_O; molecular weight: 306.40) is the most abundant molecule among the alkaloids of *G. elegans* [11] and its chemical structure is shown in Figure 1 [12]. Previous studies reported that koumine has shown promise as an anti-tumor, anxiolytic, and analgesic agent [13,14,15]. Moreover, koumine was demonstrated to have a significant analgesic effect in rodent behavioral models of inflammatory and neuropathic pain [16].

In the present study, we investigated the protective effect of koumine on LPS-induced apoptosis in RAW 264.7 macrophages. Here we provide evidence for the first time that koumine prevented lipopolysaccharide-induced apoptosis through regulation of mitochondrial pathway in RAW 264.7 macrophages.

## 2. Results

### 2.1. Cell Viability

**Figure 2:**
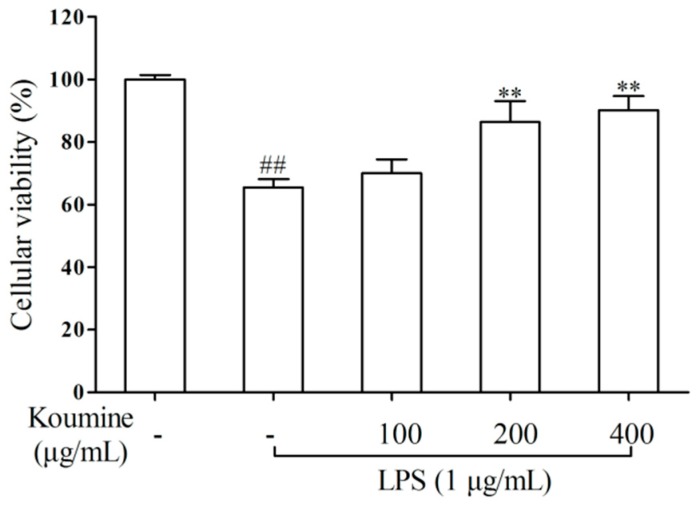
Protective effect of koumine on viability of RAW 264.7 macrophages measured by the MTT assay. Cells were pretreated with or without different concentrations of koumine (100, 200 and 400 μg/mL) for 1 h, and exposed to 1 μg/mL of LPS for 18 h in the mediums containing koumine. Then cell viability was determined by MTT assay. The data were expressed as mean ± SD (*n* = 5). ^##^ *p* < 0.01 vs. control group; ** *p* < 0.01 vs. LPS group. - means non-koumine treatment.

MTT assay was performed to determine the suppressive effect of koumine in RAW 264.7 macrophages. As shown in Figure 2, a reduction in cell viability from 100% to 67.6% was observed after incubation with 1 μg/mL of LPS for 24 h (*p* < 0.01). However, when it was pretreated with koumine, a marked increase in cell viability was observed in the LPS-stimulated RAW 264.7 cells.

### 2.2. iNOS qRT-PCR Assay

**Figure 3:**
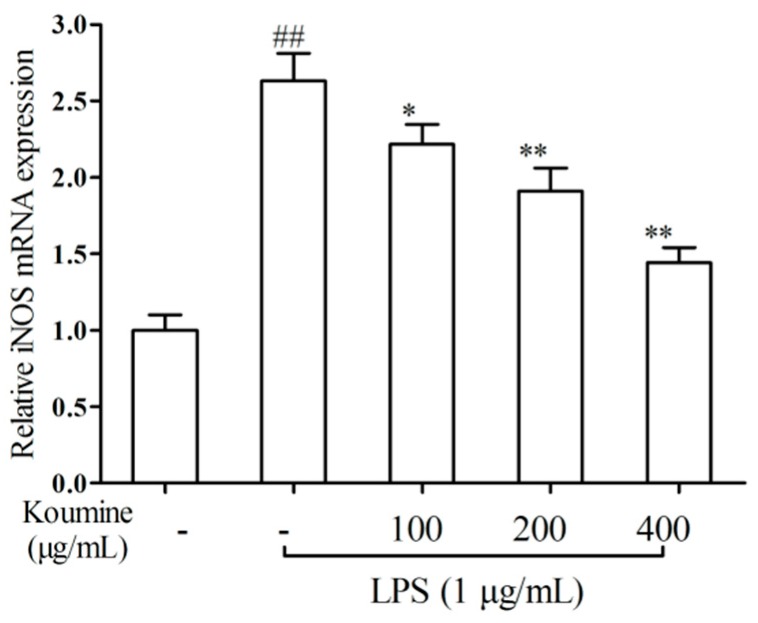
Suppression of the levels of iNOS by koumine in LPS-mediated RAW 264.7 macrophages. Cells were precultured with koumine (100, 200 and 400 μg/mL) for 1 h, and exposed to 1 μg/mL of LPS for 18 h in the mediums containing koumine. The level of iNOS and β-actin was determined using qRT-PCR assay. The level of iNOS expression was calculated after normalizing signals against the “housekeeping” gene β-actin. The data were expressed as mean ± SD. (*n* = 3). ^##^ *p* < 0.01 vs. control group; * *p* < 0.05 and ** *p* < 0.01 vs. LPS group. - means non-koumine treatment.

We investigated the effect of koumine on LPS-stimulated the levels of *iNOS* mRNA. As shown in Figure 3, the level of *iNOS* mRNA was obviously reduced after the cells were cultured with LPS in the medium containing koumine, compared with LPS only group. In LPS-stimulated RAW 264.7 cells, the inhibitory effect of koumine on *iNOS* mRNA level was showed at a concentration of 100 μg/mL, and the maximum effect of koumine was evident with a concentration of 400 μg/mL.

### 2.3. ROS Assay

**Figure 4:**
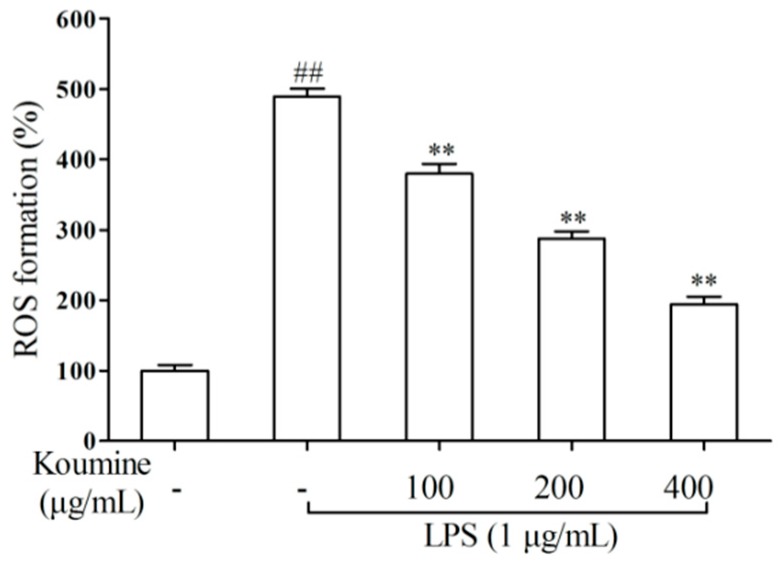
Koumine precultivation suppressed LPS-induced production of ROS in RAW 264.7 macrophages. Cells were precultured with koumine (100, 200 and 400 μg/mL) for 1 h, and exposed to 1 μg/mL of LPS for 18 h in the mediums containing various concentrations of koumine. The analysis of ROS levels was determined by DCFH-DA after treatment. The data were expressed as mean ± SD. (*n* = 3). ^##^ *p* < 0.01 vs. control group; ** *p* < 0.01 vs. LPS group. - means non-koumine treatment.

To examine if the protective effects of koumine are due to a decrease of ROS production in LPS-mediated RAW 264.7 cells, the production of ROS was determined by using dichlorofluorescein-diacetate (DCFH-DA). RAW 264.7 cells treated with LPS rapidly increased intracellular ROS level, which was effectively attenuated by pretreatment with koumine in a dose-dependent manner (Figure 4).

### 2.4. TNF-α, IL-1β, and IL-6 mRNA Expression

**Figure 5:**
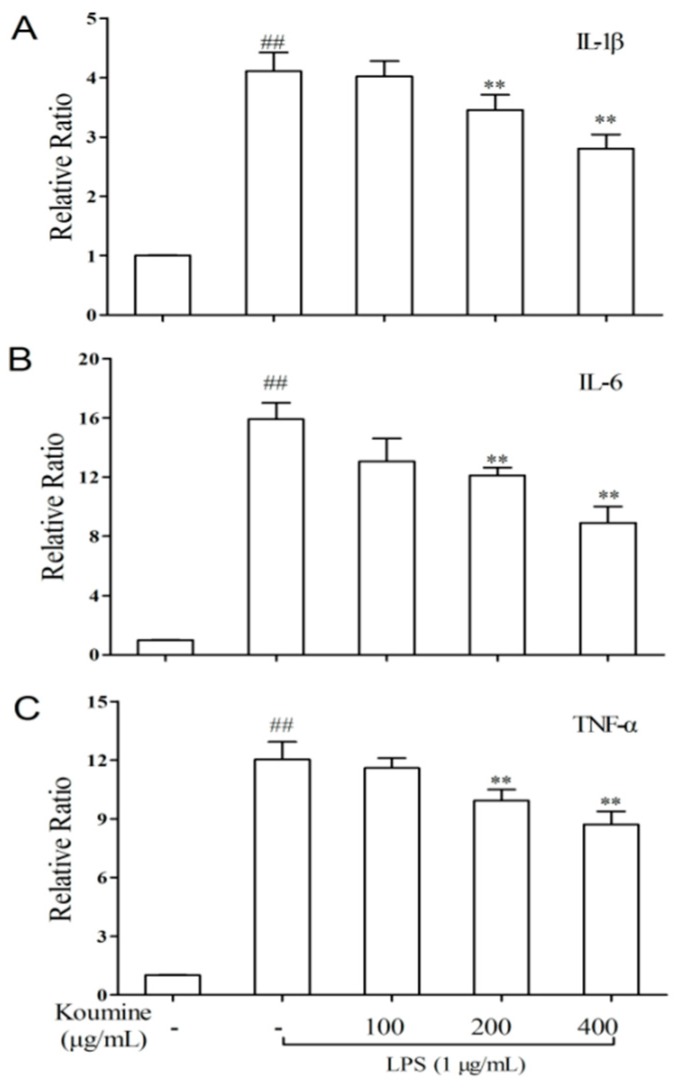
Koumine precultivation hampered LPS-induced expressions of inflammatory cytokine mRNAs in RAW 264.7 macrophages. Cells were precultured with koumine (100, 200 and 400 μg/mL) for 1 h, and exposed to 1 μg/mL of LPS for 18 h in the mediums containing koumine. The analysis of IL-1β (**A**); IL-6 (**B**); and TNF-α (**C**) mRNA expressions in the cells were determined by qRT-PCR. The levels of IL-1β, IL-6, and TNF-α expression were calculated after normalizing signals against the “housekeeping” gene β-actin, respectively. The data were expressed as mean ± SD. (*n* = 3). ^##^ *p* < 0.01 vs. control group; ** *p* < 0.01 vs. LPS group. - means non-koumine treatment.

Quantification of inflammatory cytokine mRNAs was analyzed by quantitative real-time polymerase chain reaction (qRT-PCR) in the macrophages. An obvious promotion in the expression of TNF-α, IL-1β, and IL-6 mRNAs was exhibited in macrophages treated with LPS only. Additionally, koumine concentration dependently prevents the expressions of IL-1β (Figure 5A), IL-6 (Figure 5B), and TNF-α (Figure 5C) mRNA in LPS-induced RAW 264.7 cells. The minimum inhibitory concentration was showed at a concentration of 200 μg/mL koumine, and the maximum inhibitory concentration was at a concentration of 400 μg/mL koumine.

### 2.5. Mitochondrial Injury and Cell Apoptosis

**Figure 6:**
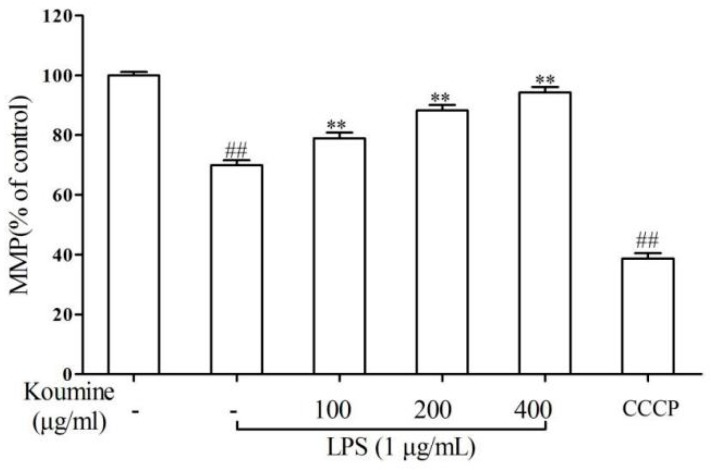
Effects of koumine on mitochondrial membrane potential in LPS-induced RAW 264.7 macrophages. Cells were pretreated with 100~400 μg/mL koumine for 1 h, and then treated with LPS (1 μg/mL) for 18 h in the preincubation mediums. JC-1 dye was used to measure ΔΨm using a fluorescence microplate reader. The ΔΨm of RAW 264.7 cells in each group were calculated as the fluorescence ratio of red to green. Cell treatments with 40 μM carbonylcyanidem–chlorophenyl-hydrazone (CCCP) were used as positive control. The data were expressed as mean ± SD. (*n* = 3). ^##^ *p* < 0.01 vs. control group; ** *p* < 0.01 vs. LPS group. - means non-koumine treatment.

**Figure 7:**
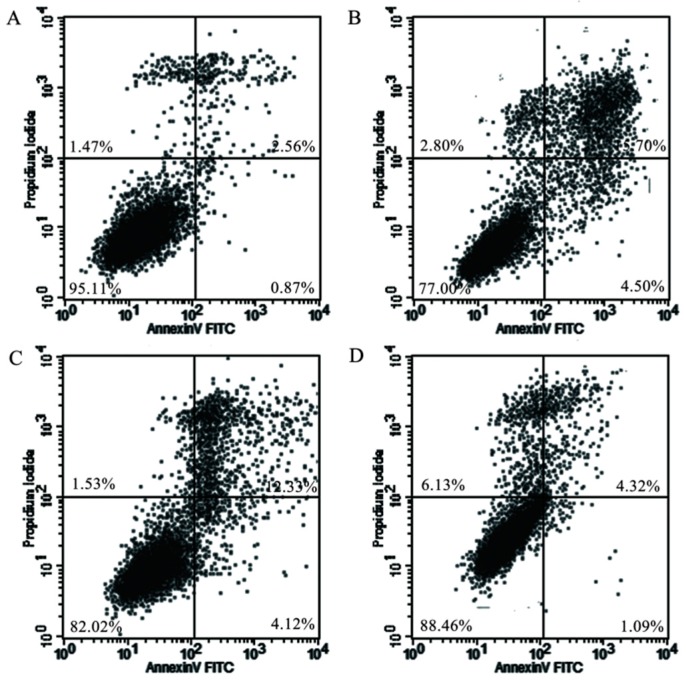

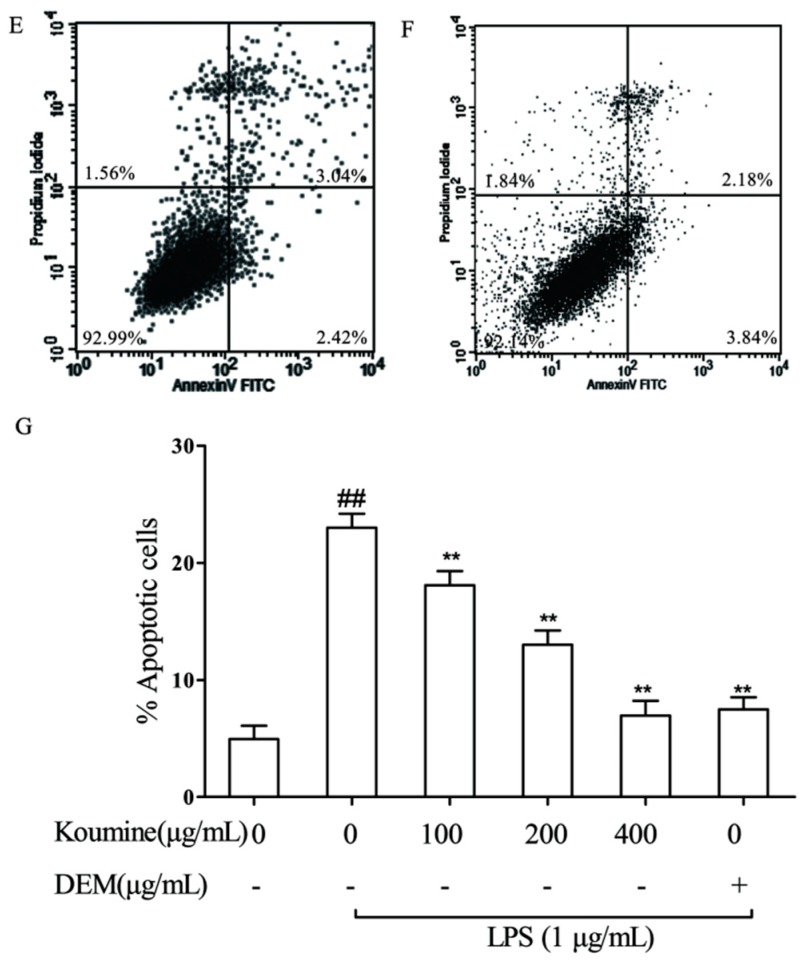
Effects of koumine on LPS-stimulated apoptosis in RAW 264.7 cells measured by flow cytometry. (**A**) Control; (**B**) 1 μg/mL LPS; (**C**–**E**) cells were precultured withkoumine (100, 200, and 400 μg/mL) or 10 μg/mL dexamethasone (**F**) for 1 h, respectively, followed by the incubation of 1 μg/mL LPS for 18 h; (**G**) koumine dose-dependently reduced the apoptotic rates of RAW 264.7 cells induced by LPS. Cells incubated with 1 μg/mL LPS for 19 h were used as a positive controls. Data are expressed as the mean ± SD. (*n* = 3). ^##^ *p* < 0.01 vs. control group; ** *p* < 0.01 vs. LPS group. - means non-dexamethasone treatment, + means 10 μg/mL dexamethasone treatment.

The results showed that LPS treatment deteriorated mitochondrial permeability of RAW 264.7 macrophages, whereas cells exposed to koumine restored mitochondrial membrane potential (ΔΨm), as shown in Figure 6. In order to confirm cellular apoptotic rate, the apoptotic rates were measured by flow cytometry. The results indicated that RAW 264.7 cells without any treatments did not showed obvious apoptosis (Figure 7A). In addition, LPS treatment significantly induced RAW 264.7 cell apoptosis (Figure 7B), whereas apoptotic cell death rate was dose-dependently decreased (*p* < 0.05) by pretreatment with 100, 200, and 400 μg/mL of koumine (Figure 7C–F).

### 2.6. Analysis of Caspase Activities

**Figure 8:**
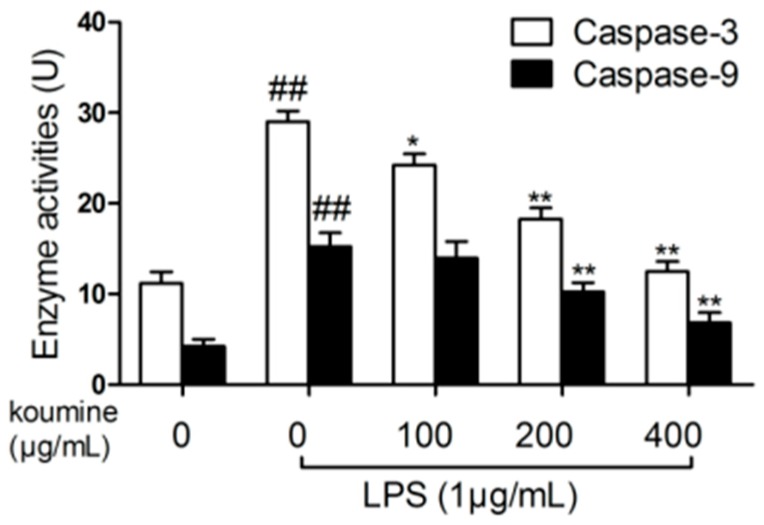
Effects of koumine on caspase activities in LPS-induced RAW 264.7 macrophages. Cells were pretreated with 100–400 μg/mL koumine or 10 μg/mL dexamethasone (DEM) for 1 h, and then treated with 1 μg/mL LPS for 18 h in the preincubation mediums. Caspase activities were measured by caspase-3 and -9 activity assay kits. The data were expressed as mean ± SD. (*n* = 3). ^##^ *p* < 0.01 vs. control group; * *p* < 0.05 and ** *p* < 0.01 vs. LPS group.

As shown in Figure 8, our result showed that caspase-9 and -3 activities were significantly higher in LPS-stimulated cells compared to control cells. Koumine pretreatment dose-dependently inhibited caspase-9 and -3 activities mediated by LPS. The maximum inhibitory effect was at a concentration of 400 μg/mL koumine.

### 2.7. Regulation of Apoptosis-Related Proteins

**Figure 9:**
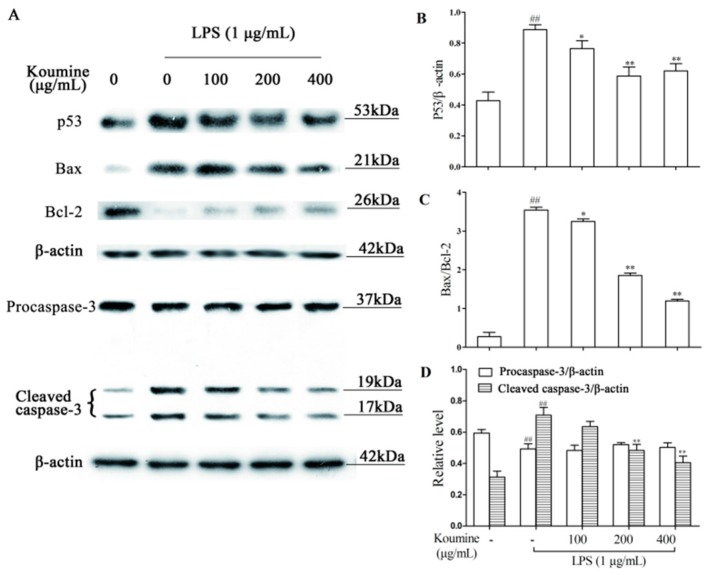
Effects of koumine on apoptosis-related proteins in LPS-induced RAW 264.7 macrophages. Cells were pretreated with 100~400 μg/mL koumine for 1 h, and then treated with 1 μg/mL LPS in the preincubation mediums for 18 h. The apoptosis-related proteins including Bax, Bcl-2, p53, and caspase-3 were measured by Western bolt. β-actin are used as internal controls. (**A**) The expressions of p53, Bax, Bcl-2, and caspase-3 proteins; (**B**) the quantification histogram of p53 protein level; (**C**) the ratio of Bax/Bcl-2 protein level; (**D**) the quantification histogram of caspase-3 protein level. The data were expressed as mean ± SD. (*n* = 3). ^##^ *p* < 0.01 vs. control group; * *p* < 0.05 and ** *p* < 0.01 vs. LPS group. - means non-koumine treatment.

We determined the expressions of p53, Bcl-2, Bax, and caspase-3 proteins associated with cell apoptosis. As shown in Figure 9, exposure of RAW 264.7 cells to 1 μg/mL LPS led to increase levels of p53, Bax, and cleaved caspase-3 proteins. Meanwhile, the expression of the anti-apoptotic Bcl-2 protein was reduced by LPS treatment. Pretreatment of with 100, 200, and 400 μg/mL koumine attenuated LPS-stimulated the levels of p53 protein (Figure 9B), Bax/Bcl-2 (Figure 9C), and cleaved caspase-3 (Figure 9D). These results suggested that koumine exhibited a protective effect for the LPS-induced RAW 264.7 cells.

## 3. Discussion

This study explored whether koumine protected against LPS-mediated apoptosis in RAW 264.7 macrophages. Furthermore, we revealed the mechanism underlying the koumine protection to LPS-induced apoptosis in RAW 264.7 macrophages. Our study proved that koumine has the ameliorative effect in LPS-mediated apoptosis in a macrophage model. The regulation occurs likely through a mitochondrial caspase-dependent pathway.

ROSs—including hydrogen peroxide (H_2_O_2_), hydroxyl radical (OH^−^), and superoxide anion (O^2−^)—are physiologically produced at a basal rate. Much evidence supports that a ROS-dependent oxidative stress response is an important mechanism involved in the innate immune response activated by LPS. Previous studies indicated that there is a close connection between oxidative stress machinery and inflammatory signaling [17]. Excessive oxidative stress leads to an unavoidable consequence of inflammatory reaction—even in serious intact cells—and tissue injury [18]. It has been proven that LPS administration stimulates production of hydroxyl radical in the early phase of inflammation and damages brain tissue after a longer exposure to LPS [19]. A growing body of experimental work indicates that oxidative stress mediated by LPS is associated with the pathogenesis of inflammatory diseases. Therefore, suppression of LPS-induced oxidative stress was regarded as a key strategy for inflammatory disease prevention. Botanical extracts, especially alkaloids, have been shown to possess anti-oxidative and anti-inflammatory activities. It has been well established that *Taraxacum officinale* Weber extracts suppressed oxidative stress and inflammatory responses via promotion of de novo synthesis of antioxidative enzymes and inhibits the expression of iNOS [20]. Alkaloids exacted from *Chelidonium majus* exhibited significant inhibitory effect on the LPS-mediated the production of NO and the induction of iNOS in RAW 264.7 macrophage cells, suggesting that those alkaloids prevented the iNOS-mediated NO syntheses at the transcriptional level [21]. Differing with these studies, Lee et al. found that 13 alkyl-substituted berberine alkaloids concentration-dependently suppressed NO production in LPS-treated splenic macrophages, but did not affect TNF-α protein/mRNA expression and *iNOS* mRNA expressions [22]. In this study, the results showed that preculture with koumine obviously reduced ROS release, iNOS level, suppressed the mRNA expressions of inflammatory cytokines (TNF-α, IL-1β, and IL-6), and restored cell growth in LPS-stimulated RAW 264.7 cells, indicating that koumine exerts antioxidative and inflammatory activities. The results are different with Wang et al. who assessed the effects of koumine on the CD4^+^ T cell growth [23]. They demonstrated 20–200 mg/mL koumine significantly reduced cell growth and IL-2 level in ConA- or PHA-mediated murine lymphocytes as compared with the controls due to its immunosuppressive effect and reduction of IL-2 secretion. Numerous studies indicate that CD4^+^ T lymphocytes and macrophages regulate the release of different types of cytokines which exert different function. T cells and IL-2 pays a viral role in stimulating immune response and inhibiting autoimmunity. CD4^+^ T cells could differentiate into effector Th1 cells which predominantly secrete IL-2, IL-12, and interferon-gamma (IFN-γ) to take part in cell-mediated inflammatory reactions [24,25,26]. Moreover, IL-2 receptors (IL-2Rs) stimulate proliferation of stimulated CD4^+^, CD8^+^, CD4^−^, CD8^−^, and γδ subsets of lymphocytes with maintenance of functional activity [27,28]. However, using IL-2, T cells can be expanded in vivo which in turn perform strong immunosuppression [29]. High-dose IL-2 as a treatment for people with cancer might product toxic reactions, like a vascular leak in the spleen, liver, or lungs [30,31], and low dose IL-2 could help inhibiting immunosuppression after transplantation [32]. On the other hand, some cytokines such as TNF-α, IL-1β, and IL-6 regulate the differentiation of various cells including macrophages in the immune system via various inflammatory pathways to trigger immune reaction as presented in this study. Thus, we can deduce that koumine might play a dual regulatory role in innate immunity.

Apoptosis is a type I form of programmed cell death that is induced by various intracellular stimuli, including oxidative stress, bacteria invasion, and DNA damage, via outer mitochondrial membrane permeabilization [33]. Mitochondria—unique organelles for energy transformation—are crucial for cellular proliferation, regulation of the cellular redox state, as well as cell apoptosis. The mitochondria respiratory chain is a major site of ROS production in the cells, and mitochondria are particularly vulnerable to oxidative stress after mitochondrial dysfunction, depolarization of mitochondrial membrane potential (ΔΨm), and the opening of the mitochondrial permeability transition pore complex has been induced. Furthermore, the expressions of pro- and anti-apoptotic Bcl-2 family proteins which belong to the intrinsic mitochondrial apoptotic pathway are changed. Subsequently, various caspases are stimulated and cell apoptosis is irreversible, indicating that ROS as mediators produced by the mitochondria appear to be associated with early and late steps of the regulation of cell apoptosis [34,35]. In some cells, the anti-apoptotic Bcl-2 family proteins prevent activations of caspases at their upstream to prevent apoptosis [36]. It was evident from Fleury et al. [37] that suppression of apoptosis through anti-apoptotic Bcl-xL and Bcl-2 is relative to a reduction of the cellular redox potential and/or a protection against ROS. Macrophages make major contributions to the homeostasis of the host and inflammatory immunopathology. They rapidly respond to invasion to promote immune responses and conversely, repair injured tissue in the aftermath of an immune response by downmodulating inflammatory responses [38]. Rapid efferocytic apoptosis of macrophages with effective clearance could inhibit proinflammatory response, such as production of large amounts of mediators (TNF-α, IL-1, and IL-6), nitric oxide, and reactive oxygen intermediaries. The action is aimed at decrease of lesion size and cellularity. In advanced lesions, the apoptotic macrophages are not efficiently cleared by efferocytosis. The apoptotic macrophages that accumulate eventually undergo secondary necrosis, because they are too much to clear out [39]. In this study, we determined that LPS significantly increased RAW 264.7 cell apoptosis. We also found that LPS decreased mitochondrial membrane potential, promoted activations of caspase-3 and caspase-9, increased expressions of Bax and cleaved caspase-3 and decreased level of Bcl-2 protein in RAW 264.7 cells, suggesting that LPS likely induced apoptosis through caspase-dependent mitochondrial death pathway. This result is in agreement with results already obtained by Langford et al. [40]. However, koumine pretreatment reversed this effect. The data showed that RAW 264.7 macrophages pretreated with koumine suppressed the LPS-stimulated mitochondrial apoptotic pathway including structural mitochondrial damage, decrease of expression of Bcl-2 protein, activation of caspase-9 and -3, and increase of expressions of Bax and caspase-3 proteins.

The nuclear transcription factor p53 can control the intrinsic pathway of apoptosis related to mitochondria. A host of various p53-induced genes including PERP, Bax, IGF-BP3, and Fas/Apo-1 were proposed to play a role in p53-mediated cell death [41]. P53-mediated transcription-independent apoptosis needs to involve Bax, mitochondrial cytochrome c release and caspase activation in the case nucleus is absence, indicating that p53 can activate the apoptotic program directly from the cytoplasm [42]. In the present study, pretreatment with koumine reduced LPS-induced activation p53, suggesting koumine alleviated apoptosis through regulation of p53 pathway, which results in the decrease of mitochondrial membrane potential, suppression of caspase cascade, and—ultimately—amelioration of cell apoptosis.

## 4. Materials and Methods

### 4.1. Materials

The koumine was purechased from Shanghai Kang Biao chemicals Co., LTD (≥98% purity; Shanghai, China). LPS (from *E. coli*, isotype 0111:B4) was purchased from Sigma (St. Louis, MO, USA, Cat# L2630-10MG, Lot# 114M4009v). A 1 mg/mL stock solution of LPS was prepared in cell culture medium and further diluted to a final concentration of 1 μg/mL. Caspase-3 and -9 activity assay kits were purchased from Beyotime (Nanjing, China). Primary antibodies against Bax, Bcl-2, p53, and β-actin were from BD Pharmingen (San Diego, CA, USA), Primary antibodies against caspase-3 was purchased from CST (Danvers, MA, USA). Polyvinylidene fluoride (PVDF) membrane and Amersham ECL Advance Western blot detection kits were obtained from GE Heathcare Life (Logan, UT, USA).

### 4.2. Cell Culture

Murine macrophage RAW 264.7 cells were obtained from Conservation Genetics CAS Kunming Cell Bank, and subcultured to confluence in Dulbecco’s modified Eagle’s medium (DMEM; Gibco, GrandIsland, NY, USA) containing 100 U/mL penicillin (HyClone, Logan, UT, USA), 10% (*v*/*v*) fetal bovine serum (Gibco, GrandIsland, NY, USA), and 100 μg/mL streptomycin (HyClone, Logan, UT, USA) in a humidified 5% CO_2_ incubator at 37 °C.

### 4.3. MTT Assay

Cell cytotoxicity was evaluated by MTT (3-(4,5-Dimethylthiazol-2-yl)-2,5-Diphenyltetrazolium Bromide) assay. For cell cytotoxic assay, RAW 264.7 cells (3 × 10^4^ cells per well in 96-well culture plates) were incubated for 24 h, or pre-incubated with 100, 200, or 400 μg/mL koumine for 1 h after 1 μg/mL LPS treatment for 18 h. MTT (the final concentration of 0.5 mg/mL) was added for 4 h. Subsequently, dimethyl sulfoxide (DMSO) was added to dissolve the formazan crystals after removing the culture medium. Data was read immediately at 570 nm. The optical density of formazan formed in control was taken as 100% of cell viability. Samples were measured in quintuplicate, and the experiment was repeated three times.

### 4.4. Determination of ROS

The level of intracellular ROS was determined by a fluorometric assay using the ROS sensitive fluorescent dye DCFH-DA (Beyotime, Nanjing, China) as the probe. The prepared RAW 264.7 macrophages (1 × 10^6^ cells/well) were pretreated with 100, 200, and 400 μg/mL koumine for 1 h followed by 1 μg/mL of LPS exposure in the preincubation mediums for 18 h. Samples were then treated with 10 μm DCFH-DA for 30 min in the dark. After cells were washed twice with PBS, fluorescence was measured at 488 nm excitation and 525 nm emission wavelength in a SpectraMax^®^ M2 Microplate Reader (Molecular Devices, Sunnyvale, CA, USA), respectively. Samples were measured in triplicate, and the experiment was repeated three times.

### 4.5. iNOS, IL-1β, IL-6, and TNF-α qRT-PCR Assay

Cells were seeded at a density of 2 × 10^6^ cells/well in 6-well plates. After stimulation, RNA was isolated from RAW 264.7 cells using Trizol^®^ reagent (Invitrogen, Carlsbad, CA, USA) according to the manufacturer’s instructions. 1 μg of total RNA was reverse-transcribed by the cDNA Synthesis Kit (Takara, Shiga, Japan). The quantification of relative mRNA concentrations was measured by qRT-PCR using a 7500 Fast Real-Time PCR System (Applied Biosystems, Foster City, CA, USA) and the SYBR green Plus reagent kit (Roche Applied Science, Mannheim, Germany), as described elsewhere [43]. The PCR mixtures were subjected to an initial denaturation of 95 °C for 10 min, followed by 40 cycles of amplification (denaturation at 95 °C for 10 s, annealing at 59 °C for 50 s, and elongation at 72 °C for 30 s). A final elongation at 72 °C for 10 min was performed. The primer sequences were as follows: iNOS, forward (5′-AAC ATC AGG TCG GCC ATC ACT-3′) and reverse (5′-CCA GAG GCA GCA CAT CAA AGC-3′); IL-1β, forward (5′-CGT TCC CAT TAG ACA ACT GCA-3′) and reverse (5′-GGT ATA GAT TCT TTC CTT TGA GGC-3′); IL-6, forward (5′-ACG GCC TTC CCT ACT TC-3′) and reverse (5′-TTC CAC GAT TTC CCA GA -3′); TNF-α, forward (5′-TGA GGA CCA AGG AGG AAA GTA TGT-3′) and reverse (5′-CAG CAG GTG TCG TTG TTC AGG-3′); β-actin, forward (5′-CAT CCT GCG TCT GGA CCT GG-3′) and reverse (5′-TAA TGT CAC GCA CGA TTT CC-3′). Samples were measured in triplicate, and each reaction was run three times.

### 4.6. Mitochondrial Membrane Potential

Cells (5 × 10^5^ cells/well) were seeded in 12-well tissue culture plates overnight and then pretreated with 100, 200, and 400 μg/mL koumine for 1 h followed by 1 μg/mL of LPS exposure in the preincubation mediums for 18 h. Subsequently, mitochondrial membrane potential assay kit with JC-1 (Beyotime, Nanjing, China) was used to measure the mitochondrial membrane potential (△ψm) of RAW 264.7 cells according to the manufacturer’s directions. Briefly, after cells were washed with PBS twice, cells were loaded with JC-1 for 30 min at 37 °C in the dark. Following staining, JC-1 dyeing buffer was pre-cooled to 4 °C, and the cells were rinsed twice with it. Fluorescent intensity of both mitochondrial JC-1 monomers (λex 490 nm, λem 530 nm) and aggregates (λex 525 nm, λem 590 nm) were detected using SpectraMax^®^ M2 Microplate Reader (Molecular Devices, Sunnyvale, CA, USA), respectively. As a positive control, mitochondria were depolarized by treating macrophage with 40 μM carbonylcyanidem–chlorophenyl-hydrazone (CCCP) at 37 °C for 10 min. Samples were measured in triplicate, and the experiment was repeated three times.

### 4.7. Flow Cytometry

Cells were seeded in 24-well plates at a density of 0.5 × 10^6^ cells per well for 24 h. These cells were pre-incubated with koumine (100, 200 and 400 μg/mL) or 10 μg/mL dexamethasone (DEM) for 1 h after 18 h of 1 μg/mL LPS treatment. Then cell apoptosis was determined using an annexin V-fluorescein isothiocyanate (FITC) detection kit (Beyotime, Nanjing, China), as described in the protocol. Briefly, cells were trypsinized, washed in PBS, and resuspended in 195 μL in binding buffer (10 mM HEPES (hydroxyethyl piperazineethanesulfonic acid), pH 7.4, 140 mM NaCl, 2.5 mM CaCl_2_). Cell suspension (1 × 10^6^ cells/mL) was incubated with 5 μL annexin V conjugated to FITC cells and 10 μL propidiumiodide (PI) for 15 min at 25 °C without light. Cells were examined with Becton Dickinson FACScan analysis software (BD Immunocytometry Systems; BD Biosciences, Becton Dickinson, Mountain View, CA, USA). Cells incubated with 1 μg/mL LPS for 19 h were used as a positive control. Samples were measured in triplicate.

### 4.8. Caspase-3 and -9 Activities

The Caspase-3 and Caspase-9 Activity Kits (Beyotime, Nanjing, China) were used to measure the activities of caspase-3 and -9, which is based on spectophotometric detection of the chromophore *p*-nitroaniline (*p*NA) after cleavage from the labeled substrate acetyl-Asp-Glu-Val-Asp *p*-nitroanilide (Ac-DEVD-*p*NA) and acetyl-Leu-Glu-His-Asp *p*-nitroanilide (Ac-LEHD-*p*NA), respectively. Cells were pre-incubated with 100, 200, and 400 μg/mL koumine or 10 μg/mL dexamethasone (DEM) for 1 h after 18 h of 1 μg/mL LPS treatment, respectively. For assays, 1 × 10^6^ cells were washed with cold PBS and lysed on ice. After centrifugation at 16,000–20,000 *g* for 15 min at 4 °C, caspase were assays performed in 96-well plates by incubating 50 μL supernatant per sample with 10 μL caspase substrate (Ac-DEVD-*p*NA or Ac-LEHD-*p*NA) (2 mM) and 40 μL reaction buffer (1% NP-40, 20 mM Tris–HCl (pH 7.5), 137 mM Nacl and 10% glycerol) for 1.5 h at 37 °C. Absorbance was read at 405 nm using a microplate reader (MK3, Thermo, Waltham, MA, USA). Caspase-3 activity was expressed as the change in enzyme activity relative to untreated control cultures.

### 4.9. Western Blot Analysis

Cells (1 × 10^6^ cells/well) were seeded in 6-well plates for 24 h, and pretreated with 100, 200, and 400 μg/mL koumine for 1 h followed by 1 μg/mL of LPS exposure in the preincubation mediums. After 18 h incubation, the cellular proteins were extracted using lysis buffer containing 1% NP-40, 150 mmol/L NaCl, 50 mmol/L Tris-HCl, pH 8.0, 0.5% sodium deoxycholate, and 0.1% SDS. The concentration of total proteins was measured using the bicinchoninic acid assay (BCA) (Pierce, Rockford, IL, USA). Equal amounts of protein samples were separated by 10% sodium dodecyl sulfate polyacrylamide gel electrophoresis, transferred onto PVDF membranes, and blocked by incubating the membrane in blocking solution containing 5% nonfat milk (Yili, Tianjin, China) for 1 h at room temperature. The PVDF membranes were washed, followed by incubation with the diluted respective primary antibodies (anti-β-actin, anti-caspase-3, anti-Bax, anti-p53, and anti-Bcl-2) at 4 °C overnight. The membrane was washed with PBST followed by incubation with the secondary antibody for 1 h at room temperature. Subsequently, the protein bands were detected using ECL reagents. Chemiluminescent signals were detected and analyzed using the ChemiDoc XRS imaging system (Bio-Rad, Hercules, CA, USA).

### 4.10. Statistical Analysis

Data were expressed as mean ± SD. Data were analyzed using LSD tests and one-way ANOVA using SPSS 19 software (Version 13.0; IBM SPSS, Inc., Chicago, IL, USA). A *p* values < 0.05 was considered statistically significant.

## 5. Conclusions

Koumine, an active alkaloid of *G. elegans*, protects RAW 264.7 macrophage against LPS-induced cell apoptosis by inhibiting intracellular ROS production and p53 activation, followed by interfering with induction of the mitochondrial caspase-dependent pathway.
